# Clinical profile and outcomes among patients with cardiac implantable electronic device presenting as isolated pocket infection, pocket-related infective endocarditis, or lead-related infective endocarditis

**DOI:** 10.1093/europace/euaf053

**Published:** 2025-03-14

**Authors:** Wojciech Jacheć, Anna Polewczyk, Dorota Nowosielecka, Andrzej Kutarski

**Affiliations:** 2nd Department of Cardiology, Faculty of Medical Sciences in Zabrze, Medical University of Silesia, Poland; Institute of Medical Sciences, Jan Kochanowski University Kielce, 5, Żeromskiego St, 25-369 Kielce, Poland; Department of Cardiac Surgery, Świętokrzyskie Cardiology Center, Kielce, Poland; Department of Cardiology, The Pope John Paul II Province Hospital, Zamość, Poland; Department of Cardiac Surgery, The Pope John Paul II Province Hospital, Zamość, Poland; 2nd Department of Cardiology, Faculty of Medical Sciences in Zabrze, Medical University of Silesia, Poland

**Keywords:** Isolated pocket infection, Pocket infection complicated by infective endocarditis

## Abstract

**Aims:**

The clinical spectrum of cardiac implantable electronic device (CIED) infections includes isolated pocket infection (IPI), pocket infection complicated by infective endocarditis (PIRIE), and lead-related infective endocarditis (LRIE). The aim of this study was to assess the risk factors, clinical course, and outcomes in patients with CIED infections and to demonstrate differences between PIRIE and LRIE.

**Methods and results:**

The retrospective analysis of data from 3847 patients undergoing transvenous lead extraction for non-infectious (2640; 68.62%) and infectious (1207; 31.38%) indications, including 361 (29.91%) IPI, 472 (39.11%) PIRIE, and 374 (30.99%) LRIE, showed some differences in risk factors, clinical course, and outcomes between the subgroups. Unlike PIRIE, diabetes [hazard ratio (HR) = 1.488; 95% confidence interval (CI; 1.178–1.879), *P* < 0.001] and lead abrasion [HR = 2.117; 95% CI (1.665–2.691), *P* < 0.001] increased the risk of LRIE. The risk of pocket infection spread was greater with *Staphylococcus aureus* infection [HR = 1.596; 95% CI (1.202–2.120), *P* < 0.001]. Compared with LRIE, patients with PIRIE had lower levels of inflammatory markers and lower prevalence of vegetations. Mortality in PIRIE compared with LRIE patients was lower (53.18 vs. 62.30%; *P* < 0.001) and comparable to IPI (50.69%; *P* = 0.162) at long-term [median 1828 (815–3139) days] follow-up.

**Conclusion:**

Cardiac implantable electronic device infections share common risk factors; however, diabetes and intra-cardiac lead abrasion predispose to LRIE, whereas multiple leads and *S. aureus* in pocket culture are risk factors for pocket infection spread. Compared with LRIE, the clinical course of PIRIE was milder, and short- and long-term mortalities were lower, but comparable with IPI after >1 year. This may be an argument in favour of categorization into primary LRIE and secondary endocarditis, i.e. PIRIE.

## Graphical Abstract

**Graphical Abstract:**
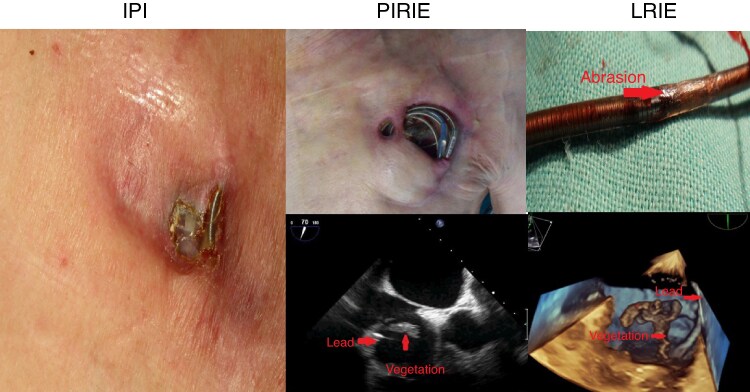

**What’s new?**

- This study identifies, for the first time, differences in factors predisposing to cardiac implantable electronic device (CIED) infections, including isolated pocket infection, pocket infection-related infective endocarditis (PIRIE), and lead-related infective endocarditis (LRIE).
- Diabetes and intra-cardiac lead abrasions were the leading causes of LRIE, less frequently observed in pocket infection complicated by infective endocarditis.
- The clinical course of CIED infection and long-term prognosis in patients with infective endocarditis due to pocket infection are much more favourable than in the case of isolated endocarditis. This argues in favour of categorization into primary LRIE and secondary pocket infection-dependent endocarditis.

## Introduction

**Figure 1:**
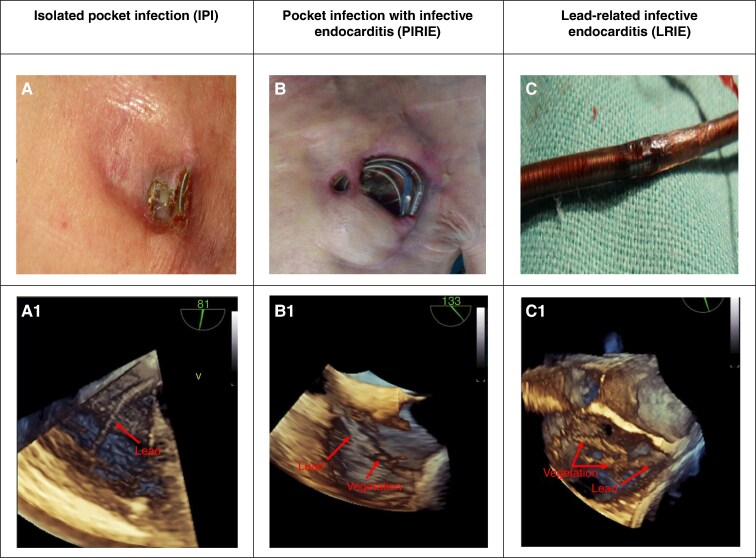
Types of CIED infections. (*A*) Isolated pocket infection with pocket erosion. (*B*) Pocket infection with pocket necrosis and erosion. (*C*) Lead abrasion (lead abrasion—intra-lead darkening results from the presence of ‘old’ haemolyzed blood inside the lead. It is the first step toward bacterial colonization and infection process with the formation of vegetations). (*A*1) No vegetations on the lead. (*B*1) Small vegetations on the lead. (*C*1) Large vegetations on the lead. CIED, cardiac implantable electronic device; IPI, isolated pocket infection; LRIE, lead-related infective endocarditis; PIRIE, pocket infection-related infective endocarditis.

Infection is a serious complication affecting from 0.9 to 2.1% of patients with cardiac implantable electronic devices (CIEDs).^1–4^ The clinical spectrum of CIED infections includes isolated pocket infection (IPI), pocket infection complicated by infective endocarditis (PIRIE), and lead-related infective endocarditis (LRIE; *Figure 1*).

Risk factors associated with CIED-related infections are divided into patient dependent, device dependent, and procedure dependent.^2–7^ The most common patient-related risk factors are age, diabetes, renal failure, atrial fibrillation, immunosuppression therapy, and chronic obstructive pulmonary disease. Multiple leads, implantable cardioverter defibrillator (ICD) or cardiac resynchronization therapy (CRT) systems, and multiple CIED-related interventions are the most important device-dependent risk factors for acquiring an infection. Procedural risk factors include absence of antibiotic prophylaxis, limited operator inexperience, procedure duration, and haematoma.^2^ Cardiac implantable electronic device infections are associated with high morbidity and mortality in 10–30% of patients, more frequently in patients with infective endocarditis.^2–4,8–10^

The aim of this study was to determine which factors predispose to the occurrence of a specific type of infection: IPI, PIRIE, and LRIE, and to determine the impact of infectious complications on the clinical course and long-term survival following transvenous lead extraction (TLE).

## Methods

We performed a retrospective analysis using data from 3847 patients undergoing TLE for non-infectious and infectious indications at three high-volume TLE centres between March 2006 and March 2023.

### Definition

Pocket infection was defined as an infection of the generator pocket with local signs of inflammation.^11–13^

Abrasion caused by friction between leads was identified in the operating theatre without additional instruments. Abrasion was defined as exposure of the underlying metal conductor (perforation of silicone tube). Mild and moderate abrasions (without prolonged exposure of the conductor) were not considered in this study; abrasions in the pocket or in the subclavian region were not taken into consideration, either. The criteria for identifying friction abrasion were as follows: (i) fluoroscopic image showing dynamic continuous contact between the leads in their intra-cardiac segments (movement in opposite directions); (ii) extraction of the lead without damage; (iii) macroscopic examination revealing abrasion of the outer insulation in the intra-cardiac part of the lead (usually in the atrial or tricuspid annulus region) with exposure of the metal conductor, colour change, or possible serous or purulent effusion (dark, usually black colour) under the outer insulation; and (iv) typical form of outer casing defect with larger circumference outside and smaller circumference inside. Leads that were damaged during removal were excluded from analysis. Typical signs of damage were cracks in the silicone sheath with sharp, uneven edges with unchanged colour of the metal conductor and only the presence of red blood under the sheath at the site of lead damage.^14^

Lead-related infective endocarditis was defined according to the 2015 and 2023 ESC guidelines and the European Heart Rhythm Association (EHRA) consensus document as an infection involving leads, cardiac valve leaflets, or endocardial surface, and was diagnosed based on the Novel 2019 International CIED Infection Criteria.^11–13^

Indications for TLE and procedure effectiveness were defined according to the HRS consensus (2009 and 2017) and EHRA guidelines (2018).^15–17^

Major complications were defined as those that posed an immediate threat to life or resulted in death. Minor complications were defined as adverse events that required medical intervention, including minor procedural interventions, but did not significantly affect patient functioning.^15,16^

### Dataset and statistical methods

#### Creation of subgroups for analysis

The entire group of 3847 patients was divided into four subgroups: (i) patients with non-infectious indications for TLE (NIP; *n* = 2640; 68.62%); (ii) patients with IPI (*n* = 361; 9.38%); (iii) patients with PIRIE (*n* = 472; 12.27%); and (iv) patients with LRIE (*n* = 374; 9.73%).

We conducted a comparison of patients undergoing TLE due to non-infectious causes and because of IPI, PIRIE, and LRIE. The IPI, PIRIE, and LRIE groups were further compared with respect to risk factors, clinical course, and long-term mortality.

### Statistical methods

Depending on non-linear distribution, all continuous variables are presented as median with first and third quartiles (Q1–Q3). Missing data are described as ‘incomplete data’ in tables with the group size specified. For better understanding, data with the same median and comparable lower and upper quartile values are presented additionally as mean ± standard deviation. The categorical variables are presented as numbers and percentages. First, the significance of differences between the groups was determined using the non-parametric Pearson *χ*^2^ test or the Kruskal–Wallis analysis of variance (ANOVA) test, as appropriate. Next, variables with *P*-values <0.05 on Pearson or ANOVA tests were tested separately using the Mann–Whitney *U* test or *χ*^2^ test with or without Yates correction, as appropriate. To identify the factors predisposing to infectious CIED complications, univariable and multivariable proportional Cox regression hazard models were used. All variables (avoiding highly correlated data) with *P*-values <0.05 on univariable analysis were entered into the multivariable model. Because the components of the PADIT score and Charlson co-morbidity index were analysed separately, both indexes were excluded from the multivariable model. Time from the last non-infectious CIED-related procedure to TLE was included in the analysis. In order to assess the impact of specific infectious CIED-related complications on mortality, the Kaplan–Meier survival curves were plotted, the course of which was assessed using the log-rank test. A *P*-value <0.05 was considered statistically significant. Statistical analysis was performed with Statistica 13.3 (TIBCO Software Inc.).

### Approval of the Bioethics Committee

The study was approved by the Bioethics Committee at the Regional Chamber of Physicians in Lublin no. 288/2018/KB/VII and was carried out in accordance with the ethical standards of the 1964 Declaration of Helsinki.

## Results

Considering age at first CIED implantation [64.00 (55.00–72.00) years] and during TLE [72.00 (63.00–79.00) years], patients with PI (IPI and PIRIE) were older than non-infectious counterparts [60.00 (48.00–68.00) and 68.00 (58.50–76.00) years] and those with LRIE [62.00 (52.00–70.00) and 69.00 (59.00–77.00) years], *P* < 0.001.

Lead-related infective endocarditis subjects were more likely to have higher creatinine concentrations (1.20 vs. 1.07 mg/dL; *P* < 0.001) and more advanced heart failure symptoms (New York Heart Association class: 2.07 vs. 1.80; *P* < 0.001) compared with PIRIE patients. Patients with PIRIE less often had atrial fibrillation compared with the IPI group (22.88 vs. 29.92%), whereas diabetes mellitus was more common but on borderline statistical significance (24.79 vs. 19.94%; *P* = 0.079; *Table 1*).

**Table 1:** Clinical data of the study group

	NIP	IPI	PIRIE	LRIE	
	Group 1 *n* = 2640 *n* (%) Median (Q1–Q3) Mean ± SD^a^	Group 2 *n* = 361 *n* (%) Median (Q1–Q3) Mean ± SD^a^	Group 3 *n* = 472 *n* (%) Median (Q1–Q3) Mean ± SD^a^	Group 4 *n* = 374 *n* (%) Median (Q1–Q3) Mean ± SD^a^	ANOVA/Pearson’s *χ*^2^
Age (during TLE) (years)	68.00 (58.00–76.00)	72.00 (63.00–78.00) *P* (vs. 1) < 0.001	72.00 (62.00–80.00) *P* (vs. 1) < 0.001 *P* (vs. 2) = 0.869	69.00 (59.00–77.00) *P* (vs. 1) = 0.132 *P* (vs. 2) = 0.003 *P* (vs. 3) = 0.001	<0.001
Age (at first CIED implantation) (years)	60.00 (48.00–68.00)	64.00 (56.00–71.00) *P* (vs. 1) < 0.001	64.00 (55.00–73.00) *P* (vs. 1) < 0.001 *P* (vs. 2) = 0.887	62.00 (52.00–70.00) *P* (vs. 1) = 0.005 *P* (vs. 2) = 0.006 *P* (vs. 3) = 0.006	<0.001
Female	1123 (42.54)	96 (26.59) *P* (vs. 1) < 0.001	141 (29.87) *P* (vs. 1) < 0.001 *P* (vs. 2) = 0.286	113 (30.21) *P* (vs. 1) < 0.001 *P* (vs. 2) = 0.314 *P* (vs. 3) = 0.988	<0.001
Coronary artery disease	1461 (55.34)	208 (57.62)	267 (56.57)	194 (51.87)	0.413
Non-ischaemic cardiomyopathy	346 (13.11)	49 (13.57)	71 (15.04)	66 (17.65)	0.096
Permanent atrial fibrillation	563 (21.33)	108 (29.92) *P* (vs. 1) < 0.001	108 (22.88) *P* (vs. 1) = 0.449 *P* (vs. 2) = 0.020	97 (25.94) *P* (vs. 1) = 0.040 *P* (vs. 2) = 0.233 *P* (vs. 3) = 0.293	<0.001
Diabetes mellitus	476 (18.03)	72 (19.94) *P* (vs. 1) = 0.377	117 (24.79) *P* (vs. 1) < 0.001 *P* (vs. 2) = 0.079	109 (29.14) *P* (vs. 1) < 0.001 *P* (vs. 2) = 0.003 *P* (vs. 3) = 0.131	<0.001
Renal dysfunction (eGFR < 60 mL/min)	498 (18.88)	82 (22.78) *P* (vs. 1) = 0.079	118 (25.05) *P* (vs. 1) = 0.002 *P* (vs. 2) = 0.426	147 (39.30) *P* (vs. 1) < 0.001 *P* (vs. 2) < 0.001 *P* (vs. 3) < 0.001	<0.001
Creatinine concentration (mg/dL)	1.00 (0.85–1.22)	1.10 (0.90–1.30) *P* (vs. 1) < 0.001	1.07 (0.90–1.31) *P* (vs. 1) = 0.001 *P* (vs. 2) = 0.856	1.20 (1.00–1.70) *P* (vs. 1) < 0.001 *P* (vs. 2) < 0.001 *P* (vs. 3) < 0.001	<0.001
Artificial heart valves, annuloplasty	163 (6.17)	19 (5.26)	17 (3.60)	26 (6.95)	0.117
Immunosuppression (any, past/present)	31 (1.17)	4 (1.12) *P* (vs. 1) = 0.884	14 (2.97) *P* (vs. 1) = 0.009 *P* (vs. 2) = 0.113	15 (4.01) *P* (vs. 1) < 0.001 *P* (vs. 2) < 0.025 *P* (vs. 3) = 0.523	<0.001
Body mass index (kg/m^2^)	27.73 (25.01–30.32)	28.06 (25.83–29.06)	28.06 (25.43–29.41)	27.73 (24.80–30.02)	0.770
Long-term anticoagulation	1041 (39.43)	145 (40.17)	166 (35.17)	160 (42.78)	0.144
Long-term antiplatelet therapy	1091 (41.33)	158 (43.77)	209 (44.28)	158 (42.25)	0.578
PADIT score (points)	3 (1–5)	3 (1–6) *P* (vs. 1) = 0.039	4 (2–6) *P* (vs. 1) < 0.001 *P* (vs. 2) = 0.060	4 (2–6) *P* (vs. 1) = 0.008 *P* (vs. 2) = 0.668 *P* (vs. 3) = 0.124	<0.001
Charlson co-morbidity index (points)	4 (2–6)	4 (3–6) *P* (vs. 1) = 0.003	5 (3–8) *P* (vs. 1) < 0.001 *P* (vs. 2) = 0.116	5 (3–10) *P* (vs. 1) < 0.001 *P* (vs. 2) = 0.052 *P* (vs. 3) = 0.569	<0.001
NYHA functional class	2 (1–2) 1.83 ± 0.67^a^	2 (1–2) 1.77 ± 0.64^a^ *P* (vs. 1) = 0.129	2 (1–2) 1.80 ± 0.70^a^ *P* (vs. 1) = 0.249 *P* (vs. 2) = 0.680	2 (1–3) 2.07 ± 0.81^a^ *P* (vs. 1) < 0.001 *P* (vs. 2) < 0.001 *P* (vs. 3) < 0.001	<0.001
LVEF (%)	55.00 (37.00–61.00)	52.00 (40.00–60.00) *P* (vs. 1) = 0.087	50.00 (35.00–60.00) *P* (vs. 1) < 0.001 *P* (vs. 2) = 0.138	50.00 (35.00–60.00) *P* (vs. 1) < 0.001 *P* (vs. 2) = 0.116 *P* (vs. 3) = 0.826	<0.001

CIED, cardiac implantable electronic device; eGFR, estimated glomerular filtration rate; IPI, isolated pocket infection; LRIE, lead-related infective endocarditis; LVEF, left ventricular ejection fraction; *n*, number; NIP, non-infectious patients; NYHA, New York Heart Association; PADIT, Prevention of Arrhythmia Device Infection Trial index; PIRIE, pocket infection-related infective endocarditis; Q1, lower quartile; Q3, upper quartile; TLE, transvenous lead extraction.

^a^Mean ± SD, mean ± standard deviation (for data with the same median and comparable lower and upper quartile values).

Pocket infection-related infective endocarditis patients compared with the IPI group had lower haemoglobin concentrations (Hb: 12.40 vs. 13.40 g/dL), higher levels of white blood cells (WBC: 8140 vs. 7400 × 10^9^/L), erythrocyte sedimentation rates (19 vs. 16 mm), and C-reactive protein concentrations (CRP: 15.96 vs. 7.02 mg/L). Lead-related infective endocarditis compared with PIRIE patients was characterized by a higher prevalence of vegetations (83.96 vs. 58.00%), lower Hb concentrations (11.20 vs. 12.40 g/dL), higher WBC (9525 vs. 8140 × 10^9^/L), and higher CRP concentrations (55.90 vs. 15.96 mg/L).

The most common pathogens responsible for pocket infection progression to infective endocarditis were Staphylococci: aureus and epidermidis. *Table 2* shows that 75.23% (82/109) and 66.83% (135/202) of PI cases related to *Staphylococcus aureus* and *Staphylococcus* spp. infections, respectively, result in infection progression to infective endocarditis (*Table 2*).

**Table 2:** Clinical course, laboratory parameters, and culture results

	NIP	IPI	PIRIE	LRIE	
	Group 1 *n* = 2640 *n* (%) Median (Q1–Q3)	Group 2 *n* = 361 *n* (%) Median (Q1–Q3)	Group 3 *n* = 472 *n* (%) Median (Q1–Q3)	Group 4 *n* = 374 *n* (%) Median (Q1–Q3)	ANOVA/Pearson’s *χ*^2^
Symptom duration (months)	0	2 (1–5)	2 (1–6) *P* (vs. 2) = 0.013	2 (1–6) *P* (vs. 2) = 0.80 *P* (vs. 3) = 0.515	0.041
Vegetation presence	0 (0.00)	0 (0.00)	272 (58.00) *P* (vs. 2) < 0.001	314 (83.96) *P* (vs. 2) < 0.001 *P* (vs. 3) < 0.001	<0.001
Haemoglobin concentration (g/dL)	13.40 (12.20–14.50)	13.30 (12.10–14.40) *P* (vs. 1) = 0.240	12.40 (11.00–13.70) *P* (vs. 1) < 0.001 *P* (vs. 2) < 0.001	11.20 (9.70–12.80) *P* (vs. 1) < 0.001 *P* (vs. 2) < 0.001 *P* (vs. 3) < 0.001	<0.001
White blood cell count (10^9^/L)	7300 (6100–8740)	7400 (6280–8840) *P* (vs. 1) = 0.373	8140 (6610–9870) *P* (vs. 1) < 0.001 *P* (vs. 2) < 0.001	9525 (7345–12 055) *P* (vs. 1) < 0.001 *P* (vs. 2) < 0.001 *P* (vs. 3) < 0.001	<0.001
Neutrophil count (10^9^/L)	4.60 (3.59–6.00)	4.57 (3.70–6.20) *P* (vs. 1) = 0.606	5.10 (3.80–6.70) *P* (vs. 1) < 0.001 *P* (vs. 2) = 0.204	6.40 (4.55–9.35) *P* (vs. 1) < 0.001 *P* (vs. 2) < 0.001 *P* (vs. 3) < 0.001	<0.001
Erythrocyte sedimentation rate (mm/1 h)	11.00 (5.00–20.00)	16.00 (8.00–30.00) *P* (vs. 1) < 0.001	19.00 (10.00–41.00) *P* (vs. 1) < 0.001 *P* (vs. 2) = 0.051	34.00 (20.00–62.00) *P* (vs. 1) < 0.001 *P* (vs. 2) < 0.001 *P* (vs. 3) < 0.001	<0.001
C-reactive protein concentration (mg/L) (incomplete data: *n* = 3643)	*n* = 2471 2.30 (0.73–8.60)	7.02 (2.21–20.54) *n* = 343 *P* (vs. 1) < 0.001	15.96 (4.50–48.20) *n* = 466 *P* (vs. 1) < 0.001 *P* (vs. 2) < 0.001	55.90 (18.13–118.6) *n* = 363 *P* (vs. 1) < 0.001 *P* (vs. 2) < 0.001 *P* (vs. 3) < 0.001	<0.001
Procalcitonin concentration (µg/L) (incomplete data: *n* = 448)	*n* = 82 0.05 (0.04–0.11)	0.08 (0.05–0.10) *n* = 57 *P* (vs. 1) = 0.259	0.10 (0.04–0.35) *n* = 138 *P* (vs. 1) = 0.042 *P* (vs. 2) = 0.171	0.20 (0.10–1.53) *n* = 171 *P* (vs. 1) < 0.001 *P* (vs. 2) < 0.001 *P* (vs. 3) < 0.001	<0.001
Culture results: *Staphylococcus aureus*		27 (7.56)	82 (17.41) *P* (vs. 2) < 0.001	34 (9.09) *P* (vs. 2) = 0.429 *P* (vs. 3) < 0.001	<0.001
Culture results: *Staphylococcus epidermidis*		67 (18.77)	135 (28.66) *P* (vs. 2) = 0.001	74 (19.79) *P* (vs. 2) = 0.674 *P* (vs. 3) = 0.004	<0.001
Culture results: Staphylococci coagulase-negative		23 (6.44)	22 (4.67)	15 (4.01)	0.307
Culture results: other staphylococci		25 (7.00)	50 (10.62)	34 (9.10)	0.201
Culture results: *Streptococcus*		2 (0.56)	2 (0.42)	5 (3.70)	0.268
Culture results: other bacteria		19 (5.32)	28 (5.94) *P* (vs. 2) = 0.701	43 (11.50) *P* (vs. 2) = 0.002 *P* (vs. 3) = 0.004	<0.001
Culture results: negative		128 (35.85)	93 (19.75) *P* (vs. 2) < 0.001	100 (26.73) *P* (vs. 2) = 0.010 *P* (vs. 3) = 0.013	<0.001
Lack of culture or culture results		70 (19.44)	60 (12.71) *P* (vs. 2) = 0.006	69 (18.45) *P* (vs. 2) = 0.700 *P* (vs. 3) = 0.020	0.014
TEE before/during TLE (culture negative) y/n (%)	2283/357 (86.48)	106/22 (82.81)	91/2 (97.85) *P* (vs. 2) < 0.001	97/3 (97.00) *P* (vs. 2) < 0.001 *P* (vs. 3) = 0.944	<0.001
TEE before/during TLE (culture positive) y/n (%)	0/0 (0.00)	132/31 (80.98)	290/29 (90.91) *P* (vs. 2) = 0.003	199/6 (97.075) *P* (vs. 2) < 0.001 *P* (vs. 3) = 0.013	<0.001

IPI, isolated pocket infection; LRIE, lead-related infective endocarditis; *n*, number; NIP, non-infectious patients; PADIT, Prevention of Arrhythmia Device Infection Trial index; PIRIE, pocket infection-related infective endocarditis; Q1, lower quartile; Q3, upper quartile; TEE, transoesophageal echocardiography; TLE, transvenous lead extraction, y/n, yes/no.

### System-related data and transvenous lead extraction outcome

Lead-related infective endocarditis and PIRIE patients compared with the NIP group had more active leads (1.88 ± 0.65 and 1.92 ± 0.69 vs. 1.79 ± 0.62; *P* < 0.001) and were more likely to have abandoned leads (14.17%; 19.07 vs. 8.25%; *P* < 0.001). Isolated pocket infection patients (compared with the NIP group) were also more likely to have abandoned leads (12.19 vs. 8.25%; *P* = 0.022). Lead abrasion was more common in LRIE (34.22%) and PIRIE (20.13%) patients than in the NIP (17.56%) and IPI (16.24%) groups. Patients from the LRIE or PIRIE groups vs. the NIP group more often had CRT-D systems (10.96%; 11.44 vs. 5.64%) and shorter dwell times of the oldest lead (6.79; 6.88 vs. 7.33 years). Implantable cardioverter defibrillator leads were more common in the LRIE group than in the NIP group (34.22 vs. 28.22%), whereas coronary sinus leads were more frequently encountered in the LRIE group compared with the NIP, IPI and PIRIE groups (25.94 vs. 14.89, 12.47, 17.58%). The reintervention rate index was higher in the infectious groups, especially in the PI (IPI, PIRIE) compared with the NIP and LRIE groups. Cardiac implantable electronic device-related procedures other than implantation or reimplantation were more common in the IPI, PIRIE and LRIE groups compared with NIP patients: 21.88%; 22.48%; 44.11 vs. 16.21%; *P* < 0.001, respectively.

Minor complications more often occurred in LRIE compared with the remaining groups (10.70 vs. 5.42; 4.43; 6.57%), but there were no differences in the occurrence of major complications. The clinical success rate for TLE achieved in the infectious groups was lower compared with the NIP group, but complete procedural success was comparable in all groups (*Table 3*).

**Table 3:** System-related data and TLE results

	NIP	IPI	PIRIE	LRIE	
	Group 1 *n* = 2640 *n* (%) Median (Q1–Q3) Mean ± SD^a^	Group 2 *n* = 361 *n* (%) Median (Q1–Q3) Mean ± SD^a^	Group 3 *n* = 472 *n* (%) Median (Q1–Q3) Mean ± SD^a^	Group 4 *n* = 374 *n* (%) Median (Q1–Q3) Mean ± SD^a^	ANOVA/Pearson’s *χ*^2^
Number of leads in the system 71	2 (1–2) 1.79 ± 0.62^a^	2 (1–2) 1.78 ± 0.68^a^ *P* (vs. 1) = 0.949	2 (2–2) 1.88 ± 0.65^a^ *P* (vs. 1) < 0.001 *P* (vs. 2) = 0.012	2 (1–2) 1.92 ± 0.69^a^ *P* (vs. 1) < 0.001 *P* (vs. 2) = 0.018 *P* (vs. 3) = 0.918	<0.001
Number of abandoned leads 73	0 (0–0) 0.11 ± 0.38^a^	0 (0–0) 0.18 ± 0.49^a^ *P* (vs. 1) < 0.001	0 (0–0) 0.27 ± 0.61^a^ *P* (vs. 1) < 0.001 *P* (vs. 2) = 0.022	0 (0–0) 0.21 ± 0.58^a^ *P* (vs. 1) < 0.001 *P* (vs. 2) = 0.539 *P* (vs. 3) = 0.196	<0.001
Presence of abandoned lead(s)	225 (8.25)	44 (12.19) *P* (vs. 1) = 0.022	90 (19.07) *P* (vs. 1) < 0.001 *P* (vs. 2) = 0.008	53 (14.17) *P* (vs. 1) < 0.001 *P* (vs. 2) = 0.487 *P* (vs. 3) = 0.045	<0.001
Number of leads in the heart before TLE	2 (1–2) 1.90 ± 0.71^a^	2 (2–2) 1.96 ± 0.71^a^ *P* (vs. 1) = 0.149	2 (2–2) 2.15 ± 0.78^a^ *P* (vs. 1) < 0.001 *P* (vs. 2) < 0.001	2 (2–3) 2.13 ± 0.86^a^ *P* (vs. 1) < 0.001 *P* (vs. 2) = 0.011 *P* (vs. 3) = 0.415	0.006
Lead(s) abrasion	466 (17.65)	59 (16.34) *P* (vs. 1) = 0.540	95 (20.13) *P* (vs. 1) = 0.198 *P* (vs. 2) = 0.172	128 (34.22) *P* (vs. 1) < 0.001 *P* (vs. 2) < 0.001 *P* (vs. 3) < 0.001	<0.001
ICD VR DR system	596 (22.58)	72 (19.94)	88 (18.64)	87 (23.26)	0.184
CRTD system	149 (5.64)	29 (8.03) *P* (vs. 1) = 0.071	54 (11.44) *P* (vs. 1) < 0.001 *P* (vs. 2) = 0.073	41 (10.96) *P* (vs. 1) < 0.001 *P* (vs. 2) = 0.204 *P* (vs. 3) = 0.754	<0.001
SSI with A or V lead, DDD, VDD, CRTP system	1895 (71.78)	260 (72.02)	330 (69.92)	246 (65.78)	0.103
ICD lead(s) presence	745 (28.22)	101 (27.98) *P* (vs. 1) = 0.869	142 (30.08) *P* (vs. 1) = 0.366 *P* (vs. 2) = 0.547	128 (34.22) *P* (vs. 1) = 0.006 *P* (vs. 2) = 0.061 *P* (vs. 3) = 0.137	0.046
CS lead presence	393 (14.89)	45 (12.47) *P* (vs. 1) = 0.222	83 (17.58) *P* (vs. 1) = 0.136 *P* (vs. 2) = 0.031	97 (25.94) *P* (vs. 1) < 0.001 *P* (vs. 2) < 0.001 *P* (vs. 3) = 0.005	<0.001
Number of procedures before TLE	1 (1–2)	2 (1–3) *P* (vs. 1) < 0.001	2 (1–3) *P* (vs. 1) < 0.001 *P* (vs. 2) = 0.231	2 (1–2) *P* (vs. 1) = 0.184 *P* (vs. 2) < 0.001 *P* (vs. 3) < 0.001	<0.001
Dwell time of the oldest lead (years)	7.33 (4.17–12.00)	6.58 (3.17–10.33) *P* (vs. 1) < 0.001	6.88 (3.17–10.79) *P* (vs. 1) < 0.001 *P* (vs. 2) = 0.824	6.79 (3.25–10.42) *P* (vs. 1) = 0.002 *P* (vs. 2) = 0.851 *P* (vs. 3) = 0.988	<0.001
Reintervention rate index^b^	0.20 (0.14–0.32)	0.30 (0.20–0.52) *P* (vs. 1) < 0.001	0.32 (0.20–0.55) *P* (vs. 1) = 0.001 *P* (vs. 2) = 0.427	0.25 (0.17–0.44) *P* (vs. 1) < 0.001 *P* (vs. 2) < 0.001 *P* (vs. 3) < 0.001	<0.001
Last CIED-related procedure: first implantation	1415 (53.60)	153 (42.38) *P* (vs. 1) < 0.001	191 (40.47) *P* (vs. 1) < 0.001 *P* (vs. 2) = 0.544	187 (50.00) *P* (vs. 1) = 0.282 *P* (vs. 2) = 0.024 *P* (vs. 3) = 0.003	<0.001
Last CIED-related procedure: generator replacement	797 (30.19)	129 (35.73) *P* (vs. 1) = 0.002	175 (37.08) *P* (vs. 1) = 0.003 *P* (vs. 2) = 0.678	106 (28.43) *P* (vs. 1) = 0.402 *P* (vs. 2) = 0.025 *P* (vs. 3) = 0.006	0.003
Last CIED-related procedure: lead extraction/removal/replacement	113 (4.28)	15 (4.16)	13 (2.75)	21 (5.61)	0.226
Last CIED-related procedure: upgrading/revisions without lead abandonment	172 (6.52)	50 (13.85) *P* (vs. 1) < 0.001	67 (14.19) *P* (vs. 1) < 0.001 *P* (vs. 2) = 0.893	38 (10.16) *P* (vs. 1) = 0.013 *P* (vs. 2) = 0.103 *P* (vs. 3) = 0.062	<0.001
Last CIED-related procedure: procedures with lead abandonment	143 (5.42)	14 (3.88)	26 (5.51)	22 (5.88)	0.614
Other last CIED-related procedure^c^	428 (16.21)	79 (21.88) *P* (vs. 1) < 0.001	106 (22.48) *P* (vs. 1) < 0.001 *P* (vs. 2) = 0.764	165 (44.11) *P* (vs. 1) < 0.001 *P* (vs. 2) < 0.001 *P* (vs. 3) < 0.001	<0.001
Minor complications	143 (5.42)	16 (4.43) *P* (vs. 1) = 0.433	31 (6.57) *P* (vs. 1) = 0.316 *P* (vs. 2) = 0.186	40 (10.70) *P* (vs. 1) < 0.001 *P* (vs. 2) < 0.001 *P* (vs. 3) = 0.032	<0.001
Major complications	57 (2.16)	5 (1.39)	8 (1.69)	8 (2.14)	0.737
Complete clinical success	2618 (99.17)	345 (95.57) *P* (vs. 1) < 0.001	455 (96.40) *P* (vs. 1) < 0.001 *P* (vs. 2) = 0.534	351 (93.85) *P* (vs. 1) < 0.001 *P* (vs. 2) = 0.328 *P* (vs. 3) = 0.094	<0.001
Complete procedural success	2517 (95.34)	344 (95.29)	454 (96.19)	349 (93.32)	0.256

A, atrial; CIED, cardiac implantable electronic device; CRTP, cardiac resynchronization therapy pacemaker; CRTD, cardiac resynchronization therapy defibrillator; CS, coronary sinus; DDD, dual chamber pacemaker; DR, dual chamber; ICD, implantable cardioverter defibrillator; IPI, isolated pocket infection; *n*, number; NIP, non-infectious patients; LRIE, lead-related infective endocarditis; PIRIE, pocket infection-related infective endocarditis; Q1, lower quartile; Q3, upper quartile; SSI, single chamber pacemaker; TLE, transvenous lead extraction; V, ventricular; VDD, pacemaker with one atrial sensing, ventricular sensing/pacing lead; VR, single chamber.

^a^Mean ± SD = mean ± standard deviation (for data with the same median and comparable lower and upper quartile values).

^b^Reintervention rate index = ratio of the number of procedures before TLE and dwell time of the oldest lead (years).

^c^Other last CIED-related procedure: lead extraction/removal/replacement or upgrading/revisions without lead abandonment or procedures with lead abandonment.

### Cardiac implantable electronic device infection: results of univariable and multivariable Cox regression

Results of the univariable and multivariable Cox regression models to identify risk factors for infectious complications of CIED are summarized in the Supplementary material online, *Tables S1* and *S2*.

### Multivariable Cox regression analysis

The most common risk factors associated with pocket infection (isolated or complicated by IE) and LRIE were older age at first CIED-related procedure [hazard ratio (HR) = 1.034; 95% confidence interval (CI; 1.028–1.039), *P* < 0.001] for PI and [HR = 1.017; 95% CI (1.008–1.025), *P* < 0.001] for LRIE, male gender [HR = 1.736; 95% CI (1.477–2.037), *P* < 0.001] and [HR = 1.381; 95% CI (1.096–1.764), *P* = 0.007], respectively, higher creatinine concentrations [HR = 1.131; 95% CI (1.038–1.234), *P* = 0.005] and [HR = 1.354; 95% CI (1.269–1446), *P* < 0.001], use of immunosuppressive drugs (including steroids) [HR = 1.902; 95% CI (1.186–3.051), *P* = 0.008] and [HR = 3.858; 95% CI (2.270–6.558), *P* < 0.001], abandoned leads [HR = 1.821; 95% CI (1.489–2.226), *P* < 0.001] and [HR = 1718; 95% CI (1.243–2.375), *P* < 0.001], ICD leads [HR = 1.426; 95% CI (1.194–1.703), *P* < 0.001] and [HR = 2.069; 95% CI (1.583–2.705), *P* < 0.001], higher rates of reintervention [HR = 1.101; 95% CI (1.081–1.122), *P* < 0.001] and [HR = 1133; 95% CI (1.095–1.173), *P* < 0.001], and finally, CIED-related procedure other than first implantation [HR = 2.012; 95% CI (1.732–2.337), *P* < 0.001] and [HR = 1.316; 95% CI (1.057–1.646), *P* = 0.014].

Unlike IPI, the risk of LRIE increased in patients with diabetes [HR = 1.488; 95% CI (1.178–1.879), *P* < 0.001], and with higher creatinine concentrations [HR = 1.354; 95% CI (1.269–1.446), *P* < 0.001] per 1 mg/dL, immunosuppression therapy [HR = 3.858; 95% CI (2.270–6.558), *P* < 0.001], lead abrasion [HR = 2.117; 95% CI (1.665–2.691), *P* < 0.001], and CRT [HR = 1.582; 95% CI (1.157–2.164), *P* < 0. 004].

Unlike IPI and like LRIE patients, the PI group showed elevated risk of IE in association with immunosuppression therapy [HR = 2.417; 95% CI (1.402–4.167), *P* < 0.001], higher creatinine concentrations [HR = 1.198; 95% CI (1.080–1.328), *P* < 0.001] per 1 mg/dL, and diabetes albeit on borderline statistical significance [HR = 1.236; 95% CI (0.998–1.532), *P* = 0.053].

**Figure 2:**
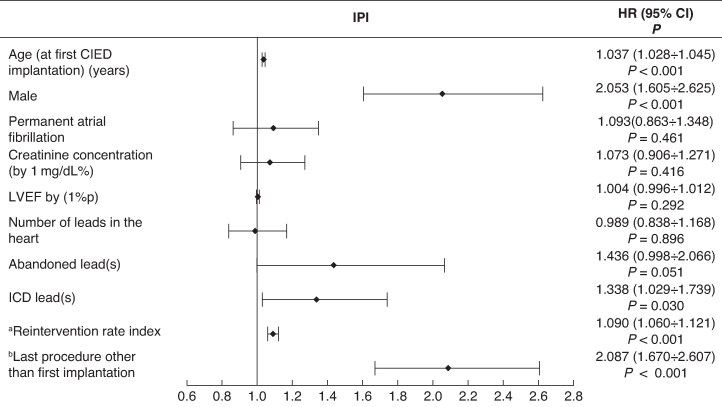
Risk factors for isolated pocket infection—multivariable Cox regression analysis. ^a^Reintervention rate index—ratio of the number of procedures before TLE to dwell time of the oldest lead (years). ^b^Last CIED-related procedure other than first implantation: lead extraction/removal/replacement or upgrading/revision without lead abandonment or procedures with lead abandonment. CIED, cardiac implantable electronic device; ICD, implantable cardioverter defibrillator; LVEF, left ventricular ejection fraction; TLE, transvenous lead extraction; 1%p, 1% point.

**Figure 3:**
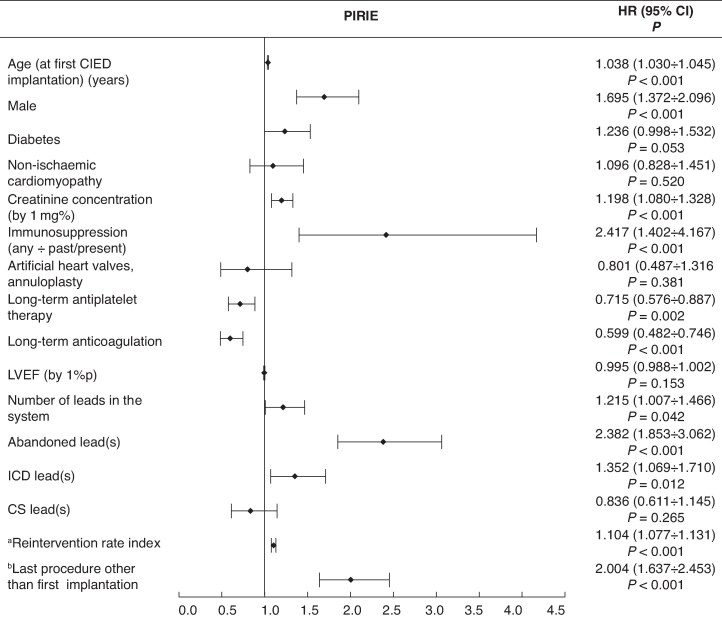
Risk factors for PIRIE—multivariable Cox regression analysis. ^a^Reintervention rate index—ratio of the number of procedures before TLE to dwell time of the oldest lead (years). ^b^Last CIED-related procedure other than first implantation: lead extraction/removal/replacement or upgrading/revision without lead abandonment or procedures with lead abandonment. CI, confidence interval; CIED, cardiac implantable electronic device; CS, coronary sinus; HR, hazard ratio; ICD, implantable cardioverter defibrillator; LVEF, left ventricular ejection fraction; NYHA FC, New York Heart Association functional class; PIRIE, pocket infection-related infective endocarditis; TLE, transvenous lead extraction; %p, per cent point.

**Figure 4:**
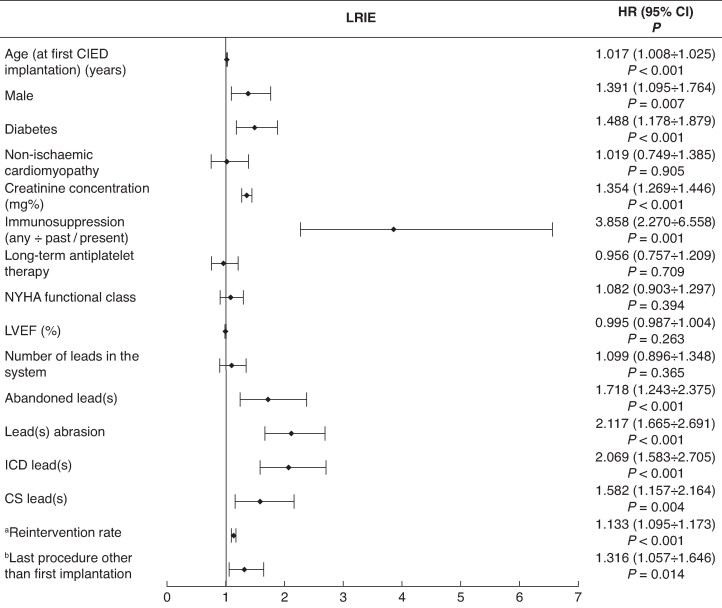
Risk factors for LRIE. ^a^Reintervention rate index—ratio of the number of procedures before TLE to dwell time of the oldest lead (years). ^b^Last CIED-related procedure other than first implantation: lead extraction/removal/replacement or upgrading/revision without lead abandonment or procedures with lead abandonment. CI, confidence interval; CIED, cardiac implantable electronic device; CS, coronary sinus; HR, hazard ratio; ICD, implantable cardioverter defibrillator; LRIE, lead-related infective endocarditis; LVEF, left ventricular ejection fraction; NYHA FC, New York Heart Association functional class; TLE, transvenous lead extraction; %p, per cent point.

The risk of pocket infection progressing to infective endocarditis increased in the presence of multiple leads in the system [HR = 1.371; 95% CI (1.146–1.641), *P* < 0.001], abandoned leads [HR = 1.371; 95% CI (1.045–1.798), *P* = 0.024], CIED with HV leads [HR = 1.510; 95% CI (1.181–1.930), *P* < 0.001], higher ratio of CIED-related procedures to dwell time of the oldest lead [HR = 1.216; 95% CI (1.154–1.218), *P* < 0.001], last procedure other than first CIED implantation [HR = 1.471; 95% CI (1.197–1.809), *P* < 0.001], and pocket infection related to *S. aureus* [HR = 1.596; 95% CI (1.202–2.120), *P* < 0.001]. The protective role of chronic antiplatelet therapy was also documented [HR = 0.715; 95% CI (0.576–0.887), *P* = 0.002] (*Figures 2–4*, Supplementary material online, *Table S2*).

### Short- and long-term survival according to indication for lead extraction

**Figure 5:**
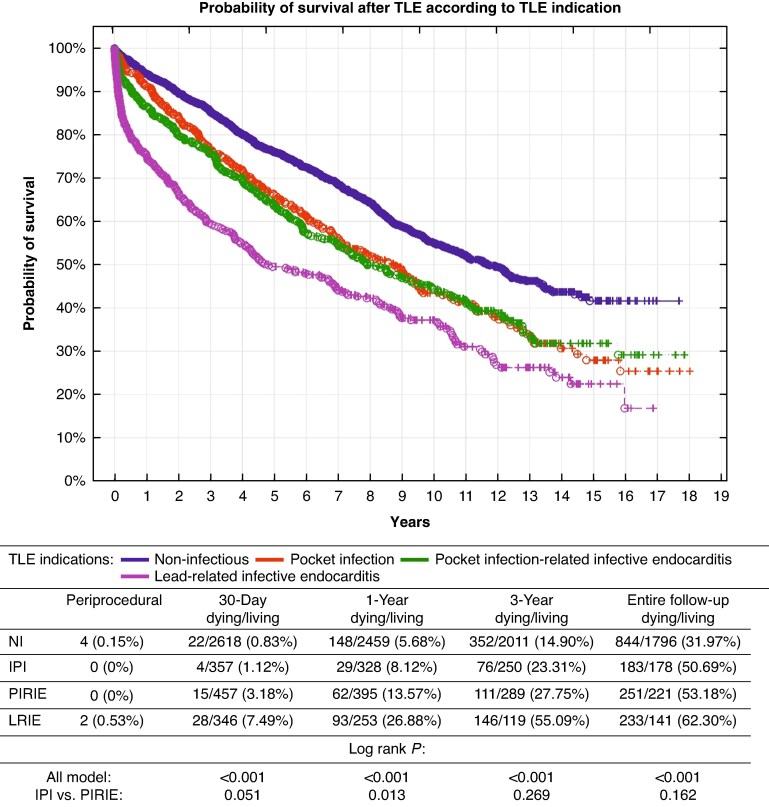
Survival after TLE according to indication for lead extraction. IPI, isolated pocket infection; LRIE, lead-related infective endocarditis; NI, non-infectious; PIRIE, pocket infection-related infective endocarditis; TLE, transvenous lead extraction.

Mean follow-up was 2072 ± 1648 days [0–6239 days (17.1 years)], median: 1828 (815–3139) days. There were six procedure-related deaths: four in the NIP group and two in the LRIE group. The patients with infectious indications for TLE compared with non-infectious patients had worse outcomes both at short- and long-term follow-up. Lead-related infective endocarditis patients showed the highest mortality at all time points. Short-term mortality in this group was nearly 10 times higher compared with mortality in non-infectious patients (7.49 vs. 0.83%; *P* < 0.001). Survival of PIRIE patients was better than that of LRIE patients (46.82 vs. 37.70%; *P* < 0.001) and worse at 1-year follow-up than in the IPI group (86.43 vs. 91.88%; *P* = 0.013), but mortality rates in IPI and PIRIE patients were comparable at longer follow-up (3-year mortality: 23.31 vs. 27.75%; *P* = 0.269 and entire follow-up mortality: 50.69 vs. 53.18%; *P* = 0.162; see Supplementary material online, *Table S3*, *Figure 5*).

## Discussion

Based on the current analysis in CIED patients, there are two different forms of endocarditis: primary LRIE and secondary infective endocarditis, i.e. PIRIE. The clinical course of PIRIE is milder than that of LRIE, and patient mortality is lower, especially in the first year after lead extraction, when it is comparable with that observed in patients with IPI.

Cardiac implantable electronic device infection can be caused by generator or lead contamination during device implantation or subsequent manipulation, direct bacterial inoculation through skin erosion, or haematogenous spread of bacteria or fungi through the bloodstream resulting from transient bloodstream infection (BSI) caused by everyday activities or invasive procedures, or sustained BSI from other sources.^18^ The most dangerous form of infection is endocarditis, which in CIED patients may occur together with a pocket infection or as isolated LRIE. The present study showed that the specific types of infections differed in risk factors, clinical course and mortality. The most common risk factors for all types of CIED infections included male gender, presence of an ICD lead, presence of an abandoned lead, last CIED-related procedure, and reintervention rate. The risk of PIRIE was higher in patients with diabetes, renal failure, immunosuppression therapy, and multiple leads. Lead abrasion was a unique risk factor for isolated LRIE. The differences in risk factors associated with the specific types of infections in the present study suggest that PIRIE and LRIE are distinct entities with different pathogenesis. In literature, there are few reports on variants of CIED-related infections. Comparison of clinical characteristics in patients with PI, PIRIE, and systemic infection in one of the recent studies did not show any significant inter-group differences in risk factors, except a higher prevalence of diabetes in patients with systemic infection.^19^

The present study showed elevated levels of inflammatory markers starting from isolated PI through PIRIE and ending with LRIE. Similar results have been found in a few previous studies.^19,20^ Other important factors that distinguish between the types of CIED-related infections include the presence of vegetations and the results of microbiological tests. In the present study, vegetations were more common in patients with LRIE compared with PIRIE. These findings are similar to previous studies.^19,20^ Blood culture results from patients in the present study showed that *S. aureus* was the most common pathogen responsible for the progression of pocket infection to infective endocarditis. The second common cause of local infection spread was *Staphylococcus epidermidis*, often found in IPI. Previous studies have also demonstrated that *S. aureus* was the main pathogen in PIRIE, whereas *Coagulase-Negative Staphylococcus* was more frequent in patients with IPI.^19,21–23^ However, lead abrasion appears to be the most important factor for differentiating the pathogenesis of IPI, PIRIE, and LRIE. Lead abrasion was documented in previous reports based on gross and microscopic examination of the leads removed during TLE.^14,24–26^ Abrasion of the outer insulation sometimes with perforation occurs as a result of intra-cardiac friction between two or more leads, but this phenomenon has also been observed in single-lead systems as a result of abrasion at the cross point of the excessive slack or rubbing against intra-cardiac structures.^24–26^ Probably due to possible conductor movements in separate channels within the lead—and especially under chronic mechanical stress at the level of the tricuspid valve or superior vena cava—the pairs of conductors are rubbing through the insulation material.^27^ Intra-cardiac abrasion of the lead favours pathogen adhesion and creates an area for pathogen colonization with formation of vegetations. An interesting observation in this study is that lead abrasion is associated with LRIE (34.22%) and PIRIE (20.13%). Although modern pacemaker leads seem to be more resistant to abrasion, an optimal lead management strategy and an optimal course of the intra-cardiac segment of the electrode can reduce the risk of LRIE.

As it is known, CIED-related infections arise from an imbalance between the human host, pathogens, and lead damage. As shown in the present analysis, the mechanism of lead abrasion (damage) is of great importance in patients with isolated LRIE.

In the current study, the 1-year mortality rate after TLE according to the type of infection was 8.1% in patients with PI, 13.6% in patients with PIRIE, and 26.9% in patients with LRIE, and was comparable with that in previous reports.^28–30^ The present study also showed that patients with PIRIE, compared with LRIE, were likely to have a milder clinical course of the disease and a better long-term prognosis (comparable with IPI at 3 years of follow-up or more) which confirms that even if PIRIE and LRIE are similar in many ways (spread of infection leading to infective endocarditis), they are different types of CIED-related infections from the perspective of pathogenesis and prognosis. These results are consistent with those presented in a recent publication.^19^ Based on the current analysis in CIED patients, we can distinguish two forms of endocarditis: primary LRIE and secondary infective endocarditis—PIRIE.

### Study limitation

The limitation of this study is that it is a retrospective analysis. Data have been collected for 17 years. Meanwhile, infection management standards have changed and gradually, but with delay, implemented in everyday clinical practice at smaller centres. On the one hand, ‘plastic’ surgery of the infected pocket was gradually abandoned, and on the other hand, intra-cardiac lead design also improved.

## Conclusions

Infectious complications associated with CIEDs (pocket infection and endocarditis) share common risk factors; however, diabetes and lead abrasion predispose to isolated lead-related endocarditis, whereas the presence of multiple leads and *S. aureus* pocket infection are risk factors for the spread of local pocket infection. Compared with LRIE, the clinical course of PIRIE was milder, and short- and long-term mortalities were lower, but comparable with IPI after >1 year. This may be an argument in favour of separating out patients with PIRIE (secondary endocarditis) from those with primary LRIE.

## Supplementary Material

euaf053_Supplementary_Data

## Data Availability

Data are available upon request with authors.
