# Supplementary material for: Clinical profile and outcomes among patients with cardiac implantable electronic device presenting as isolated pocket infection, pocket-related infective endocarditis, or lead-related infective endocarditis
Source: Europace. 2025 Mar 14;27(4):euaf053. doi: 10.1093/europace/euaf053 (PMC11982440; doi:10.1093/europace/euaf053)
Supplement: euaf053_Supplementary_Data [file euaf053_supplementary_data.docx]

**Supplementary file**

**Isolated Lead-Related Infective Endocarditis And Pocket Infection With Infective Endocarditis – Is This The Same Entity?**

Table S1. Risk factors associated with CIED infection – results of univariable regression analysis

|  | PI (all) | IPI | PIRIE | PIRIE *vs* IPI | LRIE |
| --- | --- | --- | --- | --- | --- |
|  | HR (95%CI), p | HR (95%CI), p | HR (95%CI), p | HR (95%CI), p | HR (95%CI), p |
| Age (during TLE) [years] | 1.020 (1.015÷1.025)  P < 0.001 | 1.022 (1.014÷1.030)  <0.001 | 1.022 (1.015÷1.029)  P < 0.001 | 0.995 (0.988÷1.002)  P = 0.164 | 1.009 (1.002-1.015)  P = 0.008 |
| Age (at first CIED implantation) [years] | 1.027*  (1.022÷1.032)  P < 0.001 | 1.030*  (1.022÷1.0 8)  P < 0.001 | 1.029*  (1.022÷1.035)  P < 0.001 | 1.002  (0.996÷1.099)  P = 0.478 | 1.019*  (1.012-1.026)  P < 0.001 |
| Male | 1.770* (1.522÷2.058)  P < 0.001 | 2.232* (1.621÷2.584)  P < 0.001 | 1.743* (1.431÷2.123)  P < 0.001 | 0.855 (0.702÷1.042)  P = 0.121 | 1.761* (1.410-2.193)  P < 0.001 |
| Permanent atrial fibrillation | 1.141  (0.977÷1.332)  P = 0.095 | 1.390*  (1.110÷1.742)  P = 0.004 | 1.001  (0.808÷1.241)  P = 0.990 | 0.808  (0.652÷1.003)  P = 0.053 | 1.105  (0.877-1.393)  P = 0.396 |
| Diabetes | 1.343  (1.141÷1.579)  P < 0.001 | 1.210  (0.934÷1.567)  P = 0.149 | 1.513*  (1.227÷1.865)  P < 0.001 | 1.058  (0.858÷1.304)  P = 0.601 | 1.969*  1.574-2.464)  P < 0.001 |
| Coronary artery diseases | 1.094  (0.953÷1.255)  P = 0.202 | 1.144  (0.928÷1.410)  P = 0.208 | 1.090  (0.909÷1.308)  P = 0.353 | 0.824  (0.686÷0.990)  P = 0.039 | 0.963  (0.786-1.180)  P = 0.716 |
| Non ischaemic cardiomyopathy | 1.263  (1.041÷1.533)  P = 0.018 | 1.175  0.869÷1.589)  P = 0.294 | 1.319*  (1.025÷1.599)  P = 0.032 | 1.175  (0.869÷1.589)  P = 0.294 | 1.622*  (1.243-2.119)  P < 0.001 |
| Creatinine concentration  [by 1 mg/dl  ~~%~~] | 1.238  (1.160÷1.321)  P < 0.001 | 1.292*  (1.192÷1.400)  P < 0.001 | 1.230*  (1.096÷1.379)  P < 0.001 | 1.091  (1.002÷1.188)  P = 0.046 | 1.437*  (1.359-1.520)  P < 0.001 |
| Artificial heart valves, annuloplasty | 0.764  (0.547÷1.066)  P = 0.113 | 0.879  (0.553÷1.395)  P = 0.584 | 0.614*  (0.377÷0.992)  P = 0.046 | 1.414  0.869÷2.299)  P = 0.163 | 1.119  (0.751-1.667)  P = 0.581 |
| Long-term antiplatelet therapy | 1.221  (1.064÷1.400)  P = 0.005 | 1.228  (0.997÷1.513)  P = 0.054 | 1.240*  (1.033÷1.488)  P = 0.021 | 1.044  (0.870÷1.253)  P = 0.641 | 1.266*  (1.030-1.557)  P = 0.025 |
| Long-term anticoagulation | 0.898  (0.780÷1.033)  P = 0.133 | 0.993  (0.804÷1.226)  P = 0.950 | 0.816*  (0.676÷0.986)  P÷0.035 | 0.909  (0.752÷1.099)  P = 0.326 | 1.079  (0.879-1.325)  P = 0.464 |
| Immunosuppression  (any - past / present) | 1.915  (1.200÷3.056)  P = 0.006 | 1.085  0.405÷2.908)  P = 0.871 | 2.626*  (1.543÷4.473)  P < 0.001 | 1.815  (1.066÷3.093)  P = 0.028 | 3.031*  (1.807-5.080)  P < 0.001 |
| Charlson co-morbidity Index [points] | 1.051  (1.033÷1.069)  P < 0.001 | 1.044  (1.016÷1.072)  P = 0.002 | 1.064  (1.040÷1.098)  P < 0.001 | 1.001  (0.976÷1.026)  P = 0.965 | 1.089  (1.062-1.116)  P < 0.001 |
| NYHA FC (by one) | 0.977  (0.881÷1.083)  0.656 | 0.934  0.797÷1.095)  P = 0.400 | 0.995  (0.868÷1.141)  P = 0.945 | 1.101  (0.960÷1.262)  P = 0.168 | 1.642*  (1.422-1.897)  P < 0.001 |
| LVEF by 1 %p ~~[%]~~ | 0.989  (0.984÷0.993)  P < 0.001 | 0.991*  (0.984÷0.998)  P = 0.008 | 0.986*  (0.980÷0.992)  P < 0.001 | 0.991  (0.985÷0.997)  P = 0.004 | 0.981*  (0.975-0.987)  P < 0.001 |
| PADIT score  by 1 point ~~[points]~~ | 1.087  (1.063÷1.112)  P < 0.001 | 1.070  (1.033÷1.108)  P < 0.001 | 1.190  (1.077÷1.142)  P < 0.001 | 1.057  (1.026÷1.087)  P < 0.001 | 1.087  (1.051-1.124)  P < 0.001 |
| Number of leads in the system | 1.201  (1.072÷1.354)  P = 0.002 | 1.040  (0.875÷1.237)  P = 0.655 | 1.348*  (1.140÷1.568)  P < 0.001 | 1.335  (1.148÷1.554)  P < 0.001 | 1.521*  (1.284-1.802)  P < 0.001 |
| Number of abandoned leads | 1.598  (1.420÷1.789)  P < 0.001 | 1.434  (1.166÷1.764)  P < 0.001 | 1.820  (1.578÷2.100)  P < 0.001 | 1.232  (1.062÷1.429)  P = 0.006 | 1.524  1.275-1.821)  P < 0.001 |
| Number of leads in the heart | 1.415  (1.293÷1.550)  P < 0.001 | 1.199*  (1.035÷1.386)  P = 0.015 | 1.618  (1.444÷1.813)  P < 0.001 | 1.431  (1.263÷1.621)  P < 0.001 | 1.540  (1.361-1.743)  P < 0.001 |
| Abandoned lead presence | 1.938  (1.611÷2.332)  P < 0.001 | 1.553*  (1.133÷2.129)  P = 0.006 | 2.420*  (1.923÷3.045)  P < 0.001 | 1.431  (1.135÷1.805)  P = 0.002 | 1.751*  (1.309-2.342)  P < 0.001 |
| Lead(s) abrasions | 0.924  (0.775÷1.101)  P = 0.375 | 0.802  (0.607÷1.060)  P = 0.121 | 1.009  (0.806÷1.264)  P = 0.938 | 1.106  (0.883÷1.386)  P = 0.379 | 1.830*  (1.478-2.267)  P < 0.001 |
| CRTD system | 2.660  (2.114÷3.348)  P < 0.001 | 2.322  (1.582÷3.410)  P < 0.001 | 3.124  (2.343÷4.166)  P < 0.001 | 3.124  (2.343÷4.166)  P < 0.001 | 3.832  (2.748-5.344)  P < 0.001 |
| ICD lead(s) presence | 1.420  (1.220÷1.652)  P < 0.001 | 1.375*  (1.090÷1.736)  P = 0.007 | 1.449*  (1.186÷1.769)  P < 0.001 | 1.505  (1.230÷1.840)  P < 0.001 | 2.150*  (1.726-2.679)  P < 0.001 |
| CS lead presence | 1.212  (1.004÷1.464)  P < 0.046 | 0.970  (0.709÷1.326)  P = 0.848 | 1.384*  1.091÷1.755)  P = 0.007 | 1.531  1.206÷1.924)  P < 0.001 | 2.316*  (1.835-2.922)  P < 0.001 |
| Number of procedures before TLE | 1.406  (1.340÷1.475)  P < 0.001 | 1.420  (1.315÷1.533)  P < 0.001 | 1.479  (1.390÷1.574)  P < 0.001 | 1.111  (1.039÷1.188)  P = 0.002 | 1.234  (1.131-1.347)  P < 0.001 |
| Dwell time of the oldest lead [years] | 0.925  (0.911÷0.939)  P < 0.001 | 0.917  (0.896÷0.983)  P < 0.001 | 0.926  (0.908÷0.944)  P < 0.001 | 0.956  (0.939÷0.974)  P < 0.001 | 0.891  (0.868-0.914)  P < 0.001 |
| *Reintervention ratio index | 1.112  (1.093÷1.132)  P < 0.001 | 2.234*  (2.004÷2.491)  P < 0.001 | 2.289*  (2.079÷2.521)  P < 0.001 | 1.103  (1.074÷1.133)  P < 0.001 | 1.126*  (1.089-1.163)  P < 0.001 |
| Last CIED related procedure: first implantation | 0.536  (0.466÷0.615)  P < 0.001 | 0.544  (0.442÷0.761)  P < 0.001 | 0.506  (0.421÷0.609)  P < 0.001 | 0.685  (0.570÷0.824)  P < 0.001 | 0.689  (0.562-0.854)  P < 0.001 |
| Last CIED related procedure: generator replacement | 1.450  (1.259÷1.670)  P < 0.001 | 1.425  (1.149÷1.768)  P < 0.001 | 1.497  (1.241÷1.804)  P < 0.001 | 1.308  (1.084÷1.578)  P = 0.005 | 1.082  (0.864-1.355)  P = 0.492 |
| **Other last CIED related procedure | 1.465  (1.346÷1.595)  P < 0.001 | 1.461  (1.285÷1.663)  P < 0.001 | 1.517  (1.355÷1.697)  P < 0.001 | 1.227  (1.096÷1.375)  P < 0.001 | 1.338  (1.176-1.523)  P < 0.001 |
| Last CIED related procedure: other than first implantation | 1.451  (1.184÷1.778)  P < 0.001 | 1.861*  (1.509÷2.296)  P < 0.001 | 1.973*  (1.640÷2.373)  P<0.001 | 1.456  (1.210÷1.753)  P<0.001 | 1.451*  (1.184-1.778)  P<0.001 |
| Culture results:  Staphylococcus aureus |  |  |  | *1.495  (1.177÷1.898)  P < 0.001 |  |
| Culture results:  Staphylococcus epidermidis |  |  |  | *1.289  (1.055÷1.575)  P = 0.013 |  |
| Culture results:  Staphylococci ccoagulase-negative |  |  |  | *2.167  (1.404÷3.344)  P < 0.001 |  |
| Culture results:  Other staphylococci |  |  |  | 1.096  (0.817÷1.470)  P = 0.542 |  |
| Culture results:  Streptococcus |  |  |  | 1.339  (0.333÷5.382)  P = 0.681 |  |
| Culture results:  Other bacteria |  |  |  | 1.082  (0.738÷1.586)  P = 0.686 |  |
| Culture results:  Negative |  |  |  | *0.602  (0.479÷0.756)  P < 0.001 |  |
| Lack of culture or culture results |  |  |  | 0.766  (0.583÷1.007)  P = 0.056 |  |
|  |  |  |  |  |  |

* - data included into multivariable model, NIP – non-infectious patients, IPI – isolated pocket infection,
PIRIE – pocket infection-related infective endocarditis, LRIE – lead related infective endocarditis,
TLE – transvenous lead extraction, CIED – cardiac implantable electronic devices, NYHA FC– New York Heart Association functional class, LVEF – left ventricular ejection fraction, PADIT – Prevention of Arrhythmia Device Infection Trial index, CRTD – cardiac resynchronisation therapy defibrillator, ICD – implantable cardioverter defibrillator, CS – coronary sinus, *Reintervention rate index – ratio of number of procedures before TLE and dwell time of the oldest lead [years], **Other last CIED related procedure: lead extraction / removal / replacement or upgrading / revisions without lead abandonment or procedures with lead abandonment

Table S2. Risk factors for infectious complications of CIED, results of multivariable Cox regression.

|  | PI (all) | IPI | PIRIE | PIRIE vs PI  z bakcylami | LRIE |
| --- | --- | --- | --- | --- | --- |
|  |  |  | Pocket infection related |  |  |
| Age (at first CIED implantation) [years] | 1.034  (1.028÷1.039)  P < 0.001 | 1.037  (1.028÷1.045)  P < 0.001 | 1.038  (1.030÷1.045)  P < 0.001 |  | 1.017  (1.008÷1.025)  P < 0.001 |
| Female | 0.576  (0.491÷0.677)  P < 0.001 | 0.487  (0.381÷0.623)  P < 0.001 | 0.590  (0.477÷0.729)  P < 0.001 |  | 0.719  (0.567÷0.913)  P = 0.007 |
| Male | 1.736  (1.477÷2.037  P < 0.001 | 2.053  (1.605÷2.625)  P < 0.001 | 1.695  (1.372÷2.096)  P < 0.001 |  | 1.381  (1.095÷1.764)  P = 0.007 |
| Permanent atrial fibrillation |  | 1.093  (0.863÷1.348)  P = 0.461 |  |  |  |
| Diabetes | 1.122  (0.950÷1.324)  P = 0.174 |  | 1.236  (0.998÷1.532)  P = 0.053 |  | 1.488  (1.178÷1.879)  P = 0.001 |
| CAD |  |  |  | 0.850  (0.703÷1.029)  P = 0.096 |  |
| Non ischaemic cardiomyopathy | 1.128  (0.911÷1.396)  P = 0.269 |  | 1.096  (0.828÷1.451)  P = 0.520 |  | 1.019  (0.749÷1.385)  P = 0.905 |
| Creatinine concentration  by 1 mg/dl ~~%]~~ | 1.131  (1.038÷1.234)  P = 0.005 | 1.073  (0.906÷1.271)  P = 0.416 | 1.198  (1.080÷1.328)  P < 0.001 | 1.076  (0.980÷1.180)  P = 0.124 | 1.354  (1.269÷1.446)  P < 0.001 |
| Immunosuppression  (any - past / present) | 1.902  (1.186÷3.051)  P = 0.008 |  | 2.417  (1.402÷4.167)  P < 0.001 | 1.458  (0.833÷2.552)  P = 0.286 | 3.858  (2.270÷6.558)  P < 0.001 |
| Artificial heart valves, annuloplasty |  |  | 0.801  (0.487÷1.316  P = 0.381 |  |  |
| Long-term antiplatelet therapy | 0.886  (0.762÷1.029)  P = 0.113 |  | 0.715  (0.576÷0.887)  P = 0.002 |  | 0.956  (0.757÷1.209)  P = 0.709 |
| Long-term anticoagulation |  |  | 0.599  (0.482÷0.746)  P < 0.001 |  |  |
| NYHA functional class |  |  |  |  | 1.082  (0.903÷1.297)  P = 0.394 |
| LVEF by 1 %p ~~[%]~~ | 1.000  (0.995÷1.005)  P = 0.935 | 1.004  (0.996÷1.012)  P = 0.292 | 0.995  (0.988÷1.002)  P = 0.153 | 0.998  (0.990÷1.005)  P = 0.584 | 0.995  (0.987÷1.004)  P = 0.263 |
| Number of leads in the system | 1.194  (1.042÷1.367)  P = 0.010 |  | 1.215  (1.007÷1.466)  P = 0.042 | 1.371  (1.146÷1.641)  P < 0.001 | 1.099  (0.896÷1.348)  P = 0.365 |
| Number of leads in the heart |  | 0.989  (0.838÷1.168)  P = 0.896 |  |  |  |
| Abandoned lead presence | 1.821  (1.489÷2.226)  P < 0.001 | 1.436  (0.998÷2.066)  P = 0.051 | 2.382  (1.853÷3.062)  P < 0.001 | 1.371  (1.045÷1.798)  P = 0.023 | 1.718  (1.243÷2.375)  P < 0.001 |
| Lead(s) abrasions |  |  |  |  | 2.117  (1.665÷2.691)  P < 0.001 |
| ICD lead(s) presence | 1.426  (1.194÷1.703)  P < 0.001 | 1.338  (1.029÷1.739)  P = 0.030 | 1.352  (1.069÷1.710)  P = 0.012 | 1.510  (1.181÷1.930)  P < 0.001 | 2.069  (1.583÷2.705)  P < 0.001 |
| CS lead presence | 0.705  (0.557÷0.893)  P = 0.004 |  | 0.836  (0.611÷1.145)  P = 0.265 | 0.852  (0.622÷1.166)  P = 0.316 | 1.582  (1.157÷2.164)  P = 0.004 |
| *Reintervention rate index | 1.101  (1.081÷1.122)  P < 0.001 | 1.090  (1.060÷1.121)  P < 0.001 | 1.104  (1.077÷1.131)  P < 0.001 | 1.216  (1.154÷1.218)  P < 0.001 | 1.133  (1.095÷1.173)  P < 0.001 |
| **Last CIED related procedure: other than first implantation | 2.012  (1.732÷2.337)  P < 0.001 | 2.087  (1.670÷2.607)  P < 0.001 | 2.004  (1.637÷2.453)  P < 0.001 | 1.471  (1.197÷1.809)  P < 0.001 | 1.316  (1.057÷1.646)  P = 0.014 |
| Culture results:  Staphylococcus aureus |  |  |  | 1.596  (1.202÷2.120)  P < 0.001 |  |
| Culture results:  Staphylococcus epidermidis |  |  |  | 1.195  (0.96÷1.524)  P = 0.152 |  |
| Culture results:  Staphylococci coagulase-negative |  |  |  | 1.432  (0.899÷2.281)  P = 0.131 |  |
| Culture results:  Negative |  |  |  | 0.732  (0.560÷0.955)  P = 0.022 |  |

NIP – non-infectious patients, IPI – isolated pocket infection, PIRIE – pocket infection-related infective endocarditis, LRIE – lead-related infective endocarditis, TLE – transvenous lead extraction, CIED – cardiac implantable electronic devices, NYHA FC– New York Heart Association functional class, LVEF – left ventricular ejection fraction, PADIT – Prevention of Arrhythmia Device Infection Trial index,
CRTD – cardiac resynchronisation therapy defibrillator, ICD – implantable cardioverter defibrillator, CS – coronary sinus, *Reintervention rate index – ratio of number of procedures before TLE and dwell time of the oldest lead [years], **Last CIED related procedure other than first implantation: lead extraction / removal / replacement or upgrading / revisions without lead abandonment or procedures with lead abandonment

Table S3. Short- and long-term survival after TLE according to indication for TLE

|  | Non-infectious patients  (NIP) | Isolated Pocket Infection patient  (IPI) | Pocket infection-related infective endocarditis  (PIRIE) | Lead-Related Infective Endocarditis  (LRIE) | Log rank p  (all model) |
| --- | --- | --- | --- | --- | --- |
| TLE | N=2640 | N=361 | N=472 | N=374 |  |
|  | Group 1 | Group 2 | Group 3 | Group 4 |  |
| Periprocedural deaths | 4 (0.15%) | 0 (0%) | 0 (0%) | 2 (0.53%) | <0.001 |
| 30-ty days mortality  Dead / living (%) | 22 / 2618 (0.83%) | 4 / 357 (1.12%)  P (vs 1) = 0.600 | 15 / 457 (3.18%)  P (vs 1) < 0.001  P (vs 2) = 0.051 | 28 / 346 (7.49%)  P (vs 1) < 0.001  P (vs 2) < 0.001  P (vs 3 ) = 0.004 |  |
| One-year mortality  Dead / living (%) | 148 / 2459 (5.68%) | 29 / 328 (8.12%)  P (vs 1) = 0.097 | 62 / 395 (13.57%)  P (vs 1) < 0.001  P (vs 2) = 0.013 | 93 / 253 (26.88%)  P (vs 1) < 0.001  P (vs 2) < 0.001  P (vs 3 <0.001 |  |
| Three-year mortality  Dead / living (%) | 352/ 2011 (14.90%) | 76 / 250 (23.31%)  P (vs 1) < 0.001 | 111 / 289 (27.75%)  P (vs 1) < 0.001  P (vs 2) = 0.269 | 146 / 119 (55.09%)  P (vs 1) < 0.001  P (vs 2) = 0.001  P (vs 3) = 0.001 |  |
| Entire follow-up mortality  Dead / living (%) | 844 / 1796 (31.97%) | 183 / 178 (50.69%)  P (vs 1) < 0.001 | 251 / 221 (53.18%)  P (vs 1) < 0.001  P (vs 2) = 0.162 | 233 / 141 (62.30%)  P (vs 1) < 0.001  P (vs 2) = 0.001  P (vs 3) = 0.001 |  |

TLE – transvenous lead extraction.
